# Profiles of Psychological Well-being and Coping Strategies among University Students

**DOI:** 10.3389/fpsyg.2016.01554

**Published:** 2016-10-13

**Authors:** Carlos Freire, María Del Mar Ferradás, Antonio Valle, José C. Núñez, Guillermo Vallejo

**Affiliations:** ^1^Research Group in Educational Psychology, Department of Evolutionary and Educational Psychology, University of A CoruñaA Coruña, Spain; ^2^Department of Psychology, University of OviedoOviedo, Spain

**Keywords:** psychological well-being, academic stress, coping strategies, university students, latent profile analysis

## Abstract

In the transactional model of stress, coping responses are the key to preventing the stress response. In this study, the possible role of psychological well-being as a personal determinant of coping strategies in the academic context was analyzed. Specifically, the study has two objectives: (a) to identify different profiles of students according to their level of psychological well-being; and (b) to analyze the differences between these profiles in the use of three coping strategies (positive reappraisal, support-seeking, and planning). Age, gender, and degree were estimated as covariables. A total of 1,072 university students participated in the study. Latent profile analysis was applied to four indices of psychological well-being: self-acceptance, environmental mastery, purpose in life, and personal growth. An optimal four-profile solution, reflecting significant incremental shifts from low to very high psychological well-being, was obtained. As predicted, the profile membership distinguished between participants in positive reappraisal, support-seeking, and planning. Importantly, the higher the profile of psychological well-being was, the higher the use of the three coping strategies. Gender differences in coping strategies were observed, but no interaction effects with psychological well-being were found. Age and degree were not relevant in explaining the use of coping strategies. These results suggest that psychological well-being stands as an important personal resource to favor adaptive coping strategies for academic stress.

## Introduction

In psychological research, stress is one of the variables of greatest impact due to its effect on people’s health and well-being. This evidence contrasts with the minimal attention reserved for academic stress, particularly for student stress (Michie et al., 2001), despite the fact that research has shown its high prevalence among university students (Zajacova et al., 2005; Dyson and Renk, 2006). In fact, this prevalence is comparable to that of some clinical samples (e.g., González and Landero, 2007). In this sense, high levels of stress appear to negatively affect the quality of student learning (Lumley and Provenzano, 2003) and, even more importantly, students’ physical (Loureiro et al., 2008) and psychological well-being (Garlow et al., 2008).

In this study, the role of psychological well-being as a coping resource in the academic context is analyzed. First of all, stress is defined with an emphasis on the importance of coping, taken as a modulating variable of stress responses. Subsequently, we approach the concept of psychological well-being from a eudaimonic perspective and we analyze its possible role as a personal resource to favor adaptive coping for academic demands.

According to the widely accepted transactional model of stress (see Lazarus and Folkman, 1984), stress is a dynamic interaction process between the individual and his or her environment. Therefore, the stress response does not depend solely on the existence of an environmental stressor, but also on how this stressor is perceived by the person (cognitive appraisal) and what resources and strategies he or she uses to cope (coping process).

Cognitive appraisal comprises two interdependent processes: primary appraisal and secondary appraisal. Through primary appraisal, we judge whether the situation is irrelevant, positive, or stressful. That is, that the event is irrelevant because it does not bear any implications for our well-being; positive, in that the situation is favorable for the purpose of satisfying our personal goals; or stressful in that it requires the use of resources to cope because our well-being could otherwise be at risk (stress does not have to be negative but implies the need for an adaptive effort). In turn, the stressful situation may pose a threat where, we anticipate possible damage or loss before it occurs. It may also lead to loss or damage if damage has already occurred, with consequent damage to our esteem, health, family, and social relationships, among others, and we understand that the situation will remain unchangeable. The stressful situation may also be seen as a challenge when, we consider that despite difficulties, there is a chance of profit or benefits if, we mobilize adequate resources. Thus, threat and challenge appraisals lead to different coping expectancies, since the former are associated with a lower confidence on one’s ability to cope with demands of stressful situations whereas challenge appraisals predict higher expectancies for successful coping (Skinner and Brewer, 2002). In secondary appraisal, we judge the resources at our disposal to successfully address the situation. In this process, we are aware of the discrepancy between our resources and coping strategies and the repertoire of resources and strategies required to address the stressful situation. The greater the discrepancy is, the more likely, we are to experience stress (Carver and Scheier, 1999).

Coping refers to cognitive, emotional, and/or behavioral efforts to address (master, reduce, or tolerate) a troubled person-environment relationship (Folkman and Lazarus, 1985). Accordingly, coping strategies play a crucial role in people’s health (Kraag et al., 2006), with relevant implications for subjective well-being (e.g., Parsons et al., 1996; Sheldon and Lyubomirsky, 2006; Viñas et al., 2015) and psychological well-being (e.g., Loukzadeh and Bafrooi, 2013; Portocarrero and Bernardes, 2013; Bryden et al., 2015; Mayordomo et al., 2015).

Assuming that coping strategies are important for people’s well-being, prolific research has focused on studying whether some coping mechanisms are more adaptive than others. Although the contextual nature of coping suggests that one strategy can be adaptive in one context but not in others (Endler et al., 1994), approach coping is generally considered more adaptive than avoidant coping (e.g., Gustems-Carnicer and Calderón, 2013; Syed and Seiffge-Krenke, 2015). Approach copping involves the cognitive, emotional, or behavioral strategies aimed at either resolving the stressful situation or modifying the underlying negative emotions. Conversely, avoidant coping involves the adoption of cognitive, emotional, or behavioral strategies aimed at avoiding having to deal with the problem or negative emotions that would result from the stressful situation (Endler and Parker, 1990). Based on this approach, Skinner et al. (2013), using a variety of studies as the background, comprehensively reviewed the coping procedures that proved to be effective and those that proved to be dysfunctional in the academic domain. According to their findings, the most adaptive strategies for addressing academic demands are planning, seeking instrumental support, seeking comfort (e.g., emotional support), self-support (encouraging oneself), and commitment to the tasks. However, according to the researchers, experiencing cognitive confusion, being mentally estranged from the problems, hiding the problems from people who are close, systematically blaming oneself for all evils, ruminating on the problems, and projecting the responsibility for all negative matters onto others constitute dysfunctional strategies for students, given that they hinder the completion of the task and even increase emotional distress.

The complex structure of the coping process spans the existence of a set of hierarchical categories on which coping can be conceptualized (see Skinner et al., 2003 for a review). Indeed, coping strategies constitute an intermediate category, since they represent recognizable action schemas in dealing with stressful transactions that can be expressed at the lowest level by different responses (i.e., coping behaviors) according to a specific stressful event. At the same time, coping strategies can be classified into higher order categories, called coping resources, which involve a set of bio-psycho-social resources that take part in the coping efforts by either hindering or favoring them (Cohen and Edwards, 1989) and, consequently, increasing the vulnerability or resistance to stress. Thus, these personal resources are important determinants of coping strategies (Taylor, 1991; Skinner et al., 2003). Within this set of coping resources, psychological variables are receiving increasing attention. In this sense, increasing interests exist in the study of individual strengths and potentials as optimal resources to facilitate adaptive responses to daily academic challenges and adversities, and which encompass a majority of students (e.g., Martin and Marsh, 2009; Putwain et al., 2012). This research approach is well represented by the eudaimonic well-being perspective, which posits that the maximum development of individual potential (i.e., psychological well-being) is determined by six indicators of positive psychological functioning: self-acceptance (SA), environmental mastery (EM), positive relations with others, autonomy, purpose in life (PL), and personal growth (PG; Ryff, 1989).

An extensive body of research suggests that several variables that are closely linked to these six dimensions of psychological well-being favor the adoption of adaptive coping strategies in the academic context. Some of these variables are self-esteem (Cabanach et al., 2014), perceived control (Doron et al., 2009), quality of social support (Fernández-González et al., 2015), self-determination (Ryan and Deci, 2000), PL (Freire et al., 2015), and pursuit of self-realization (Miquelon and Vallerand, 2008).

However, to date, very few studies have examined the possible role of psychological well-being, considered a global construct, as a personal resource that could favor adaptive coping to academic demands. Based on this consideration, significant differences in coping strategies have been observed in adolescent students according to their level (high vs. low) of psychological well-being (González et al., 2002; Figueroa et al., 2005). Higher levels of psychological well-being led to the adoption of adaptive strategies such as commitment, positive reappraisal, or seeking for instrumental and emotional support. Conversely, students with lower levels of psychological well-being used dysfunctional coping strategies such as ignoring the problem, blaming themselves about the situation, or taking refuge in fantastic thoughts.

González et al. (2002) and Figueroa et al. (2005) used a median split technique to determine the level of psychological well-being in their samples. This technique has the disadvantage that it dichotomizes continuous variables, which underestimates the strength of relationships and reduces statistical power for detecting true effects (Maxwell and Delaney, 1993). Such statistical limitation can be overcome by adopting a person-centered approach that groups students who have a similar functioning on psychological well-being indicators (Bhullar et al., 2014).

Therefore, the primary objective of this study is to identify profiles of psychological well-being according to their functioning in the different dimensions that comprise psychological well-being. Based on the results obtained by González et al. (2002) and Figueroa et al. (2005), we hypothesize the identification of at least two quantitative profiles consisting of students who are either low or high in indices of psychological well-being. Our second objective is to determine whether the identified profiles of psychological well-being differ in terms of coping strategies that the students adopt to deal with academic demands. It is expected that students with high functioning on psychological well-being indices use adaptive coping strategies to a greater extent than students with a profile of low psychological well-being.

Our study focused on university students, a group that has not been examined by previous research. Although from a developmental perspective the university stage typically corresponds with adolescence, some authors postulated that within the heterogeneity in this age, university students constitute a particular group. As Rodríguez and Agulló (1999) stated, the formative capital of these students partially determines a lifestyle characterized by certain values, attitudes, and life experiences that distinguish them from other young people. This set of idiosyncratic characteristics and their interaction with the learning environment may influence the students’ well-being (see Lazarus, 1999). Additionally, factors such as the transition and adaptation to the university context (Fisher, 1984), the evaluation stage (Cabanach et al., 2014), the work overload (Salanova et al., 2005), or the need for academic success that enables access to the labor market (Zeidner, 1995) contribute to stress reaching its highest point at the university stage (e.g., Dyson and Renk, 2006). All of this makes the study of the role played by psychological well-being on coping especially important among university students.

To achieve these objectives, we attempted to control for the effect of variables such as age and major, because previous studies have suggested that these variables are related to a differential use of academic stress coping strategies (e.g., Martín et al., 1997; Cassaretto et al., 2003). Controlling for the effect of gender is also important because a significant number of studies have shown that males and females use different academic coping mechanisms. While the latter would mainly choose searching for support, the former would be more likely to use some type of more direct action (Feldman et al., 2008; Matheny et al., 2008; Cabanach et al., 2013). Therefore, these three variables (age, major, and gender) could significantly affect the research results.

## Materials and Methods

### Participants

The study was conducted with students from the University of A Coruña, a small university in northern Spain with 21,362 students. Considering that, with a confidence level of 95% and a maximum margin of error of 5%, the minimum sample size required for this study was 400 subjects. Because the selection of the sample was not random, we wanted to work with a sufficiently large number of subjects so that the results would be as generalizable as possible.

Thus, a total of 1,072 students between 18 and 48 years of age (*M* = 21.09; *SD* = 3.16) participated in the study. With regard to gender, 68% (*n* = 729) were women and 32% (*n* = 343) were men. Of the total sample, 35.7% (*n* = 383) were pursuing degrees in Education Sciences (Early Childhood Education, Primary Education, Physical Education, Hearing and Language, Social Education and Speech Therapy, and Educational Psychology); 19% (*n* = 203) were pursuing degrees in the Health Sciences (Physiotherapy, Nursing, and Sciences of Physical Activity and Sport); 26% (*n* = 279) of the participants were studying technical majors (Architecture, Engineering, and Technical Architecture and Engineering of Roads, Channels, and Ports); and 19.3% (*n* = 207) were pursuing degrees in the legal and social fields (Law and Sociology). Regarding the grade variable, 28.4% of the subjects (*n* = 304) were in their first year of study; 28.6% (*n* = 307) in their second; 28.2% (*n* = 302) in their third; and 8.5% (*n* = 91) and 6.3% (*n* = 68) in their fourth and fifth years, respectively.

### Instruments

#### Psychological Well-being

The Spanish adaptation of the Ryff Scales of Psychological Well-being (Díaz et al., 2006) was used to measure psychological well-being. This instrument contains 29 items that assess the six dimensions of eudaimonic well-being proposed by Ryff (1989): SA, positive relationships with others, autonomy, EM, PL, and PG. However, previous studies with both elderly people (Tomás et al., 2012) and university students (Freire et al., 2016) have shown through confirmatory factor analysis (CFA) that the structure with the best fit included only the four dimensions constituting the core of psychological well-being (Springer and Hauser, 2006): SA (three items; e.g., “In general, I feel confident and positive about myself”); PG (four items; e.g., “I have the sense that I have developed a lot as a person over time”); EM (five items; e.g., “In general, I feel I am in charge of the situation in which I live”); and PL (six items; e.g., “I clearly understand the direction and purpose of my life”). In our study, this structure has shown a good fit to the empirical data: χ^2^/degrees of freedom (χ^2^/DF = 2.95); *p* < 0.001; goodness-of-fit index (GFI = 0.97); adjusted goodness-of-fit index (AGFI = 0.95); comparative fit index (CFI = 0.96); parsimony comparative fit index (PCFI = 0.75); Tucker Lewis index (TLI = 0.95); and root mean square error of approximation (RMSEA = 0.04). The factors for internal consistency were as follows: SA (α = 0.78), PG (α = 0.63), EM (α = 0.63), and PL (α = 0.75). The students responded to the items through a five-point Likert scale ranging from 1 (*strongly disagree*) to 5 (*strongly agree*). Higher scores reflect higher levels in each dimension of psychological well-being.

#### Coping Strategies

The instrument used to measure coping strategies was the Coping Scale of Academic Stress Questionnaire (Escala de Afrontamiento del Cuestionario de Estrés Académico, A-CEA) by Cabanach et al. (2010). The scale contains 23 items that assess three academic coping mechanisms: *positive reappraisal*, understood as a coping strategy aimed at changing the meaning of a problematic situation, highlighting its positive aspects and activating positive expectations (10 items; e.g., “When I am faced with a problematic situation, I forget unpleasant aspects and highlight the positive ones”); *support-seeking*, which involves both seeking advice and information on how to resolve a problem, and seeking understanding and support for the emotional state caused by the problem (seven items; e.g., “When I am faced with a problematic situation, I ask for advice from a family member or a close friend”); and *planning*, aimed at analyzing and designing an action plan intended to solve a problematic situation (six items; e.g., “When I am faced with a difficult situation, I list the tasks that I have to fulfill, I complete them one at a time, and I do not go to the next step until I have completed the previous one”). To contextualize the use of coping strategies in the academic context, the participants received the following written clarification at the beginning of the test: “read each item carefully and indicate to what extent you behaved accordingly when faced with an academic problematic situation.” This three-component structure has shown good psychometric properties (α between 0.81 and 0.91) in previous studies with university populations (e.g., Cabanach et al., 2009, 2013) and showed a good fit to the empirical data in the present study (χ^2^/DF = 3.74; *p* < 0.001; GFI = 0.95; AGFI = 0.94; IFC = 0.95; PCFI = 0.79; TLI = 0.95; RMSEA = 0.05) as well as adequate reliability: positive reappraisal (α = 0.86), support-seeking (α = 0.90), and planning (α = 0.81). The participant responses were collected using a five-point Likert scale ranging from 1 (*never*) to 5 (*always*).

### Procedure

The study was carried out in accordance with the recommendations of the Ethics Committee of the University of A Coruña and the American Psychological Association with written consent from all subjects in accordance with the Declaration of Helsinki. Thus, prior to participation, students were informed about the goals of the research, duration, procedure, and anonymity of their data. Participation in the study was voluntary, and students were assured that all of their responses would remain confidential and used for research purposes only. Data were collected in each of the centers attended by the students who participated in the investigation, in the classroom and during school hours. The questionnaires were administered in a single session by trained personnel.

### Data Analysis

A latent profile analysis (LPA) (Lanza et al., 2003) was performed to obtain the categorical latent variables that can group people into classes based on their characteristics. The objective of this analysis was to classify individuals from a heterogeneous population into smaller homogeneous subgroups based on individual values from numerical variables. This approach uses all of the information available in the numeric dependent variables to classify subjects into various classes using the maximum likelihood estimation method (Little and Rubin, 1987). Through this approach, the probability that an individual is correctly categorized, which enables each person to be placed in the class with best fit, is estimated simultaneously with the global model. In this study, the Mplus program version 6.11 (Muthén and Muthén, Muthén and Muthén) was used to determine which model among a finite set of models best fit the data, adding successive latent classes to the target model. As a rule, the optimum number of classes in the data sample is selected using the adjusted Lo-Mendell-Rubin maximum likelihood ratio test (LMRT) (Lo et al., 2001), the Akaike information criterion (AIC), Schwarz’s Bayesian information criterion (BIC), and the sample-size adjusted BIC (SSA-BIC), in addition to the entropy value. In this work, we also used the sample sizes for all of the subgroups as criteria.

The *p* value associated with the LMRT indicates whether the solution with more (*p* < 0.05) or fewer classes (*p* > 0.05) is the solution that best fits the data. The AIC, BIC, and SSA-BIC criteria are descriptive fit indices where lower values indicate a better fit of the model. It is desirable for these criteria to complement the information provided by the formal test of conditional fit, but the former should never replace the latter because formal testing ultimately determines the decision. Similarly, it should be noted that small classes (those containing less than 5% of the sample) are typically considered spurious classes, a condition that is often associated with the removal of an excessive number of profiles (Hipp and Bauer, 2006). Therefore, in addition to the substantive meaning of each solution, parsimony, and/or theory (i.e., structure of psychological well-being, according to Ryff, 1989) and the quality of the obtained solution, the size of the classes must also be considered to select the optimal number of classes.

To avoid biases in the standard errors as much as possible, the fact that the students were pursuing over 15 different degrees was taken into account in the data analysis. Indeed, it was expected that students completing the same degree were more homogeneous regarding their psychological well-being than those pursuing different degrees. In other words, homogeneity would be lower if the subjects were considered as independently sampled units without any relationship between them. Because the bootstrapped likelihood ratio (BLRT) is not available when using the clustering option in Mplus, the only formal test reported here is the LMRT referred to above.

Another approach considered in the assessment of the selected model concerns the analysis of the differences between classes in relation to the variables comprised in them. It was expected that the differences between classes in the criterion variables would be significant. Cohen’s *d* was used to assess the effect size.

Finally, the relationship between the profiles of psychological well-being (latent classes) and the use of academic stress coping strategies was studied through multivariate analysis of covariance (MANCOVA). As a strategy for statistical control of unwanted effects in the estimation of the type of profile of psychological well-being and the use of coping strategies, three covariates were included in the model (age, major, and gender). The three covariates were not significantly related to the explanatory variables (the type of profile of psychological well-being): age [*F*(3,1068) = 2.25, *p* > 0.05], major [*F*(3,1068) = 2.48, *p* > 0.05], and gender (Wald χ^2^ = 1.11, *p* > 0.05). The eta squared was used to calibrate the size of this relationship, by taking the dependent variables (the three coping strategies) together and individually. Cohen’s (1988) criteria were used to interpret the effect size, indicating that the effect was small when η^2^ = 0.01 (*d* = 0.20), medium when η^2^ = 0.059 (*d* = 0.50), and large when η^2^ = 0.138 (*d* = 0.80). These analyses were carried out in the SPSS 21 statistical software (IBM Corp, 2012).

## Results

### Descriptive Statistics

The descriptive statistics of the variables and Pearson correlation coefficients were performed using SPSS 21 (IBM Corp, 2012). As shown in **Table 1**, the variables were significantly inter-correlated, without presenting extreme values (the highest correlation was *r* = 0.67) and with a moderate average correlation (*r* = 0.40). The skewness and kurtosis values of the variables were within the intervals that denote a normal distribution (all were between -1 and 1).

**Table 1:** Means, standard deviations, and correlations between the four dimensions of psychological well-being and the three academic stress coping strategies (*N* = 1072).

	1	2	3	4	5	6	7
(1) Self-acceptance	-						
(2) Environmental mastery	0.65	-					
(3) Purpose in life	0.67	0.65	-				
(4) Personal growth	0.41	0.40	0.48	-			
(5) Positive reappraisal	0.51	0.38	0.41	0.24	-		
(6) Support-seeking	0.29	0.26	0.35	0.28	0.23	-	
(7) Planning	0.34	0.32	0.45	0.27	0.55	0.30	-
*M*	3.87	3.75	3.82	4.17	3.01	3.44	3.05
*SD*	0.71	0.63	0.67	0.58	0.71	0.87	0.74
*Skewness*	-0.74	-0.58	-0.64	-0.71	0.05	-0.15	0.07
*Kurtosis*	0.71	0.37	0.35	0.86	-0.46	-0.79	-0.44


*All Pearson *r* correlation coefficients are significant at *p* < 0.001. Psychological well-being scales (1 = *strongly disagree*,…5 = *strongly agree*). Coping strategies scale (1 = *never*,…5 = *always*; higher scores reflect higher levels of psychological well-being and a higher use of coping strategies).*

### Profiles of Psychological Well-being

Several models of latent profiles, including one, two, three, four, and five classes (groups), were fit to the data. Model fitting stopped when non-significant LMRT results occurred or when a group of subjects with less than 1% of the total sample was obtained. The goodness-of-fit indices of the model for each LPA are shown in **Table 2.** The shown LMRT indicated that the three-class solution provided a better fit to the data than the two-class solution (or the single-class solution). Initially, the three-class solution was deemed superior to the four-class solution, because the LMRT indicated that there were no significant differences between the two solutions (LMRT = 201.46; *p* = 0.18). However, the four-class solution was also an interesting alternative because none of the classes had a number of subjects below 5% of the total sample (size = 0). Although the AIC, BIC, and SSA-BIC criteria showed a slight decrease when comparing four classes against three (the lower they are, the better the model fit), they were not taken into account because these are nested models. However, entropy was taken into account (quality of the proposed grouping), noting that that the four-class model (entropy = 0.78) was better than the three-class model (entropy = 0.77). Therefore, after analyzing the two alternative LPA configurations (three vs. four classes), the four-class model was thought to provide the best empirical (and theoretical) fit, correctly classifying over 78% of the subjects.

**Table 2:** Results obtained when comparing the latent class models.

	Latent class models				
	
Criteria	Class 1	Class 2	Class 3	Class 4	Class 5
AIC	8411.09	7158.93	6727.65	6540.98	6506.54
BIC	8450.90	7243.55	6857.06	6715.19	6725.54
SSA-BIC	8425.49	7189.55	6774.48	6604.02	6585.79
Entropy	-	0.77	0.77	0.78	0.75
LMRT	-	1250.24	442.24	201.46	51.63
(*p*)		(0.0001)	(0.017)	(0.176)	(0.596)
Size	0	0	0	0	1


*AIC, Akaike information criterion; BIC, Bayesian information criterion; SSA-BIC, Sample-size-adjusted BIC; LMRT, Lo-Mendell-Rubin adjusted likelihood ratio test.*

**Table 3** shows the number of students in absolute (*n*) and relative (%) terms in each of the four classes of the model chosen, in addition to the classification accuracy in each class. Three classes composed most of the sample (92.5%): Class 4 with 41.9%, Class 3 with 35.4%, and Class 1 with 15.2%. However, Class 2 was somewhat particular and comprised only 7.5% of the cases. In relation to the accuracy with which subjects were classified, **Table 3** shows that Class 2 had greater classification precision (88.2%). The diagonal in **Table 3** shows the accuracy of the four classes. In line with the above, the values outside the diagonal show that individuals classified as Class 2 were the least likely to be allocated to other classes (only a 3.2% chance of being assigned to Class 3). In general, the classification accuracy of the four classes was similar and adequate.

**Table 3:** Latent class characterization and precision in the classification of individuals in each class.

		Latent class			
		
		Class 1	Class 2	Class 3	Class 4
Number of cases		163	80	379	450
Percentage of cases (%)		15.2	7.5	35.4	41.9
		
Probability of success in the classification	Class 1	**0.87**	0.00	0.00	0.13
	Class 2	0.00	**0.88**	0.12	0.00
	Class 3	0.00	0.03	**0.88**	0.09
	Class 4	0.05	0.00	0.10	**0.86**


*Class 1, very high psychological well-being; Class 2, low psychological well-being; Class 3, medium psychological well-being; Class 4, high psychological well-being. Higher scores (close to 1.00) reflect greater precision classification.*

After establishing the four-class model as the best solution, the next step was to interpret these classes. The average scores of the subjects in the latent classes, which could vary between classes and were used in this study to substantively interpret each profile, are shown in **Table 4.**

**Table 4:** Description of latent classes.

	Profiles of psychological well-being			
	
	Self-acceptance	Environmental mastery	Purpose in life	Personal growth
	
	*M* (*SE*)	*M* (*SE*)	*M* (*SE*)	*M* (*SE*)
Latent Class 1	4.65 (0.09)	4.39 (0.04)	4.63 (0.11)	4.73 (0.03)
Latent Class 2	2.50 (0.20)	2.67 (0.15)	2.54 (0.18)	3.50 (0.13)
Latent Class 3	3.54 (0.06)	3.39 (0.07)	3.46 (0.08)	3.10 (0.03)
Latent Class 4	4.12 (0.06)	4.03 (0.05)	4.07 (0.06)	4.25 (0.09)


*SE, Standard error; Latent Class 1, very high psychological well-being; Latent Class 2, low psychological well-being; Latent Class 3, medium psychological well-being; Latent Class 4, high psychological well-being. Psychological well-being scales (1 = *strongly disagree*,…5 = *strongly agree*; higher scores reflect higher levels in each dimension of psychological well-being).*

**FIGURE 1:**
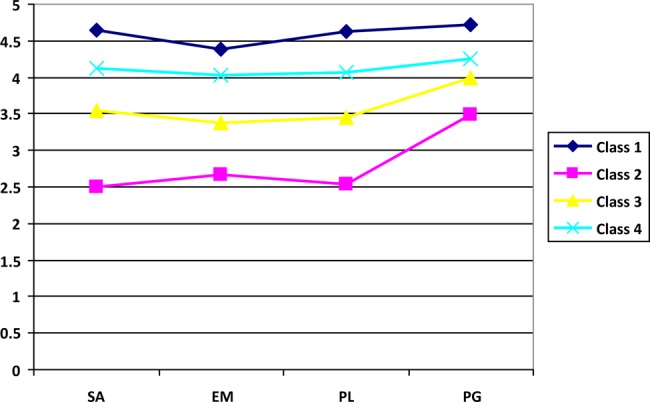
**Graphical representation of the psychological well-being profiles.** Class 1, very high psychological well-being; Class 2, low psychological well-being; Class 3, medium psychological well-being; Class 4, high psychological well-being; SA, Self-acceptance; EM, Environmental mastery; PL, Purpose in life; PG, Personal growth.

In general terms, the four classes showed similar trends in the profiles, although they had different levels in the four variables. As shown in **Figure 1**, the four profiles presented some parallelism, with greater differences between classes in the SA, EM, and PL variables and smaller differences in the PG variable. However, as shown in **Table 5**, the inter-class differences in the four variables were always significant with medium and large effect sizes (even very large in some pairs).

**Table 5:** Differences of class means across indicators of well-being.

	Indicators of psychological well-being			
	
	Self-acceptance	Environmental mastery	Purpose in life	Personal growth
Class 1 – Class 2	1.56	1.97	1.46	1.62
Class 1 – Class 3	0.99	0.81	0.81	1.23
Class 1 – Class 4	0.42	0.40	0.46	0.30
Class 2 – Class 3	0.84	0.50	0.62	0.64
Class 2 – Class 4	1.20	1.30	1.24	0.44
Class 3 – Class 4	0.49	0.53	0.46	0.18


*Class 1, very high psychological well-being; Class 2, low psychological well-being; Class 3, medium psychological well-being; Class 4, high psychological well-being. Relative size of Cohen’s *d*: negligible effect (≥-0.15 and <0.15), small effect (≥0.15 and <0.40), medium effect (≥0.40 and <0.75), large effect (≥0.75 and <1.10), very large effect (≥1.10 and <1.45), and huge effect (>1.45).*

Consequently, the four latent classes can be interpreted as *general profiles of psychological well-being* that vary in magnitude, since the four profiles displayed a similar pattern at different levels.

To qualify the four groups of students, the variable means in each class were taken as reference (see **Table 4**), in addition to the average values of the classes (Class 1 = 4.60, Class 2 = 2.80, Class 3 = 3.60, and Class 4 = 4.12) and the values of the measurement scale (1 = strongly disagree, 2 = mostly disagree, 3 = agree more than disagree, 4 = mostly agree, and 5 = strongly agree). Accordingly, Class 1 represented a *profile with very high psychological well-being*; Class 2 a *profile with low psychological well-being*; Class 3 a *profile with medium psychological well-being*; and Class 4 a *profile with high psychological well-being*.

### Differences between Profiles of Psychological Well-being in Academic Coping Strategies

The differences between profiles of psychological well-being (low, medium, high, and very high) in the use of academic coping strategies (positive reappraisal, support-seeking, and planning) was examined through various variance and covariance analyses, taking the profiles of psychological well-being (four levels) as the independent variable and the three strategies for coping with stress as dependent variables. The students’ major (the sample had 15 different majors), age (the sample was selected from the population of all of the grades in the major), and gender were incorporated as covariates. **Table 6** provides the corresponding descriptive statistics.

**Table 6:** Means and standard deviations of the profiles of psychological well-being for each of the coping strategies and their univariate tests.

	Coping strategies of academic stress					
	
Profiles of psychological well-being	Positive reappraisal		Support-seeking		Planning	
	***M***	***SD***	***M***	***SD***	***M***	***SD***
	
Low	2.42	0.66	2.90	0.89	2.43	0.71
Medium	2.74	0.61	3.21	0.83	2.82	0.65
High	3.16	0.65	3.58	0.80	3.16	0.68
Very high	3.54	0.64	3.85	0.83	3.56	0.70

**Univariate tests**	**Positive reappraisal**		**Support-seeking**		**Planning**	
	
Profiles [*F*_(3,1065)_]	93.41^∗∗^ η^2^ = 0.21		44.24^∗∗^ η^2^ = 0.11		70.91^∗∗^ η^2^ = 0.17	
Gender [*F*_(1,1065)_]	56.56^∗∗^ η^2^ = 0.05		43.76^∗∗^ η^2^ = 0.04		4.35^∗^ η^2^ = 0.004	


*Coping strategies scale (1 = *never*,…5 = *always*; higher scores reflect a higher use of coping strategies). Relative size of eta squared: negligible effect (<0.01), small effect (>0.01 and <0.059), medium effect (>0.059 and <0.138), and large effect (>0.138). ^∗^*p* < 0.05; ^∗∗^*p* < 0.001.*

Globally considered, the data suggest a statistically significant relationship between the type of profile of psychological well-being and the three coping strategies used by the students [λ_Wilks_ = 0.724, *F*(9,2587) = 40.825, *p* < 0.001, η^2^ = 0.102], with a medium effect size. The results showed significant differences between the four profiles of students with respect to the three coping strategies taken individually: positive reappraisal [*F*(3,1065) = 93.41, *p* < 0.001, η^2^ = 0.21], support-seeking [*F*(3,1065) = 44.24, *p* < 0.001, η^2^ = 0.11], and planning [*F*(3,1065) = 70.91, *p* < 0.001, η^2^ = 0.17]. In terms of effect size, the differences were large for positive reappraisal and planning and medium for support-seeking. Regarding the group means, the same trend was observed in the three dependent variables: the higher the profile of psychological well-being was, the greater the use of stress coping strategies.

With regard to the covariates included in the model, the gender variable showed significant differences in the three dependent variables: positive reappraisal [*F*(1,1065) = 56.56, *p* < 0.001, η^2^ = 0.05] and support-seeking [*F*(1,1065) = 43.76, *p* < 0.001, η^2^ = 0.04] with a medium effect size, and planning [*F*(1,1065) = 4,35, *p* < 0.05, η^2^ = 0.004], with a small effect size. The students’ major and age were not relevant in explaining the use of academic stress coping strategies.

Because gender differences were significant in the use of strategies for coping with stress, it was important to examine the possible interactions between this variable and the type of profile of psychological well-being. For this reason, a series of factorial analyses of variance were conducted, including the type of profile and gender as factors and the three stress coping strategies as dependent variables.

**FIGURE 2:**
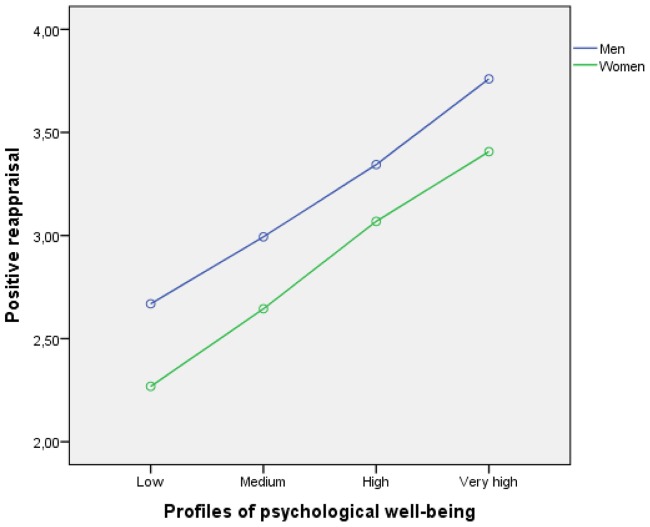
**Relationship between psychological well-being profiles (low, medium, high, and very high), use of positive reappraisal as a coping strategy, and student gender**.

**FIGURE 3:**
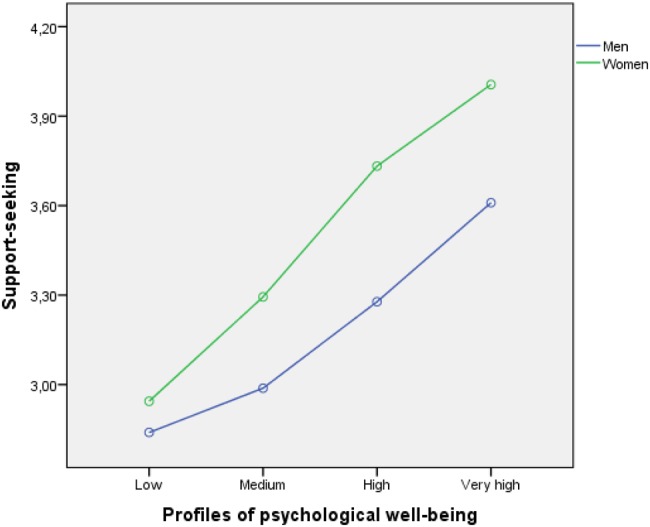
**Relationship between psychological well-being profiles (low, medium, high, and very high), use of support-seeking as a coping strategy, and student gender**.

**FIGURE 4:**
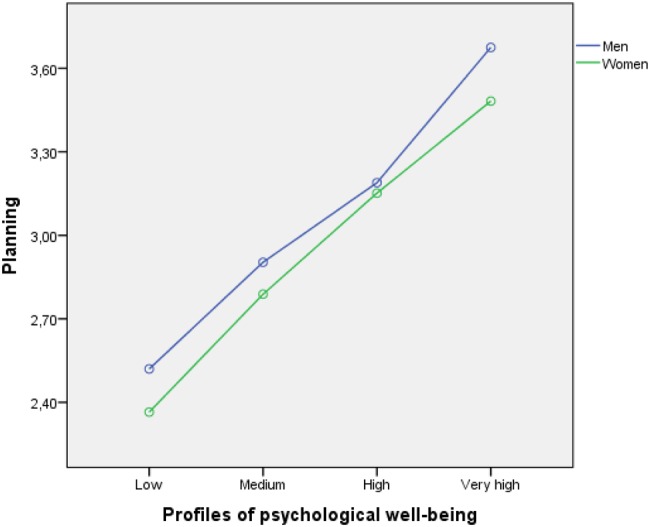
**Relationship between type of psychological well-being profile (low, medium, high, and very high), use of planning as a coping strategy, and student gender**.

The analysis results showed only the main effects on the three dependent variables. No interaction effects were found. In particular, we found that men had higher levels of positive reappraisal (see **Figure 2**) and planning (**Figure 4**) than women in the four types of profiles of psychological well-being. Such differences were similar in magnitude and yielded parallel profiles for men and women, leading to a non-interaction between the two variables (*p* > 0.05). In the case of support-seeking, women presented higher levels (see **Figure 3**). Although the gender profiles were not fully parallel, the interaction was not significant.

## Discussion

This study provides some interesting results with regard to the relationship between coping strategies and psychological well-being in a population that is especially vulnerable to stress, as is the case with university students (Zajacova et al., 2005). Previous studies on adolescents (e.g., González et al., 2002; Figueroa et al., 2005) concluded that there was a differential use of coping mechanisms depending on the individuals’ level of psychological well-being. However, in these studies, the variable-centered approach adopted to determine the level of psychological well-being failed to characterize the common profile-based patterns. To answer this question, our work adopted a person-centered approach to determine whether there were different university student profiles based on different functioning levels on several psychological well-being indicators. Additionally, we analyzed whether these profiles were significantly different in relation to the use of mechanisms including positive reappraisal, support-seeking, and planning to cope with the potentially stressful demands of the academic context.

Our initial hypothesis was based on the existence of two profiles of psychological well-being. However, this hypothesis was partially rejected, since our results identified the existence of four different profiles of university students according to their level of psychological well-being: a first group with very high psychological well-being, a second group with a high level of well-being, and two other groups with medium and low psychological well-being. Each of these profiles showed significant differences in the effect sizes of SA, EM, PL, and PG. These dimensions have been considered the core of psychological well-being (e.g., Tomás et al., 2012).

Regarding our second objective, our data suggested the existence of considerable differences between the four profiles in the use of positive reappraisal, support-seeking, and planning, evidencing that the higher the degree of SA, EM, PL, and PG reported by the students, the greater the use of these three coping strategies. These results are consistent with studies that positively relate psychological well-being and the use of adaptive coping strategies (e.g., Loukzadeh and Bafrooi, 2013; Portocarrero and Bernardes, 2013; Mayordomo et al., 2015).

The students’ major, age, and gender were considered covariates in the study. In contrast with the results obtained in other studies (e.g., Martín et al., 1997; Cassaretto et al., 2003), our findings failed to show significant differences neither for major nor age in the use of the three analyzed coping strategies. Given the breadth and diversity of majors and school years involved in our work, this finding led us to conclude that the use of adaptive coping strategies, as is the case with positive reappraisal, support-seeking, and planning, does not depend on the type of academic demands or on the students’ level of experience; instead, it depend on their own psychological functioning.

However, gender differences were significant in the use of the three coping strategies. In line with a large body of research referring to the differences between males and females in the management of academic stress (e.g., Feldman et al., 2008; Matheny et al., 2008; Cabanach et al., 2013), our results suggest that male students used positive reappraisal and planning as academic stress coping mechanisms to a greater extent than females, whereas females mainly made recourse to support-seeking.

Despite this differential use of academic coping strategies associated with gender, the findings of this study show that this variable did not significantly interact with psychological well-being in explaining the use of positive reappraisal, support-seeking, and planning. Accordingly, the positive linear trend observed in the differences between the profiles of psychological well-being in the use of the three coping strategies was almost parallel in males and females, such that in both genders, the higher the level of psychological well-being was, the greater the use of such coping mechanisms.

Overall, our data suggest that psychological well-being and, more specifically, its constitutive dimensions (SA, EM, PL, and PG) represent a personal resource of unquestionable worth to favor adaptive coping within the demands of the university context. Therefore, these findings add to the growing line of work that positively relates adaptive coping with stress and certain psychosocial variables that are closely linked to psychological well-being, such as self-esteem (Cabanach et al., 2014), hardiness (Otero-López et al., 2014), resilience (González-Torres and Artuch, 2014), PL (Freire et al., 2015), quality of social support (Fernández-González et al., 2015), and pursuit of self-realization (Park and Adler, 2003; Miquelon and Vallerand, 2008).

In summary, these results contribute to expanding the spectrum of interventions aimed at reducing student stress, addressing this issue from an eminently proactive perspective focused on the development of individual strengths and abilities. Thus, an important implication derived from these results is the need to design and implement initiatives and programs to promote students’ psychological well-being. In this regard, numerous universities from different geographical and cultural contexts have successfully developed initiatives aimed at fostering students’ personal potentials and virtues in recent years (Moshki et al., 2012; Carter et al., 2013; Romero et al., 2013).

However, it is necessary to consider some limitations in this work. First, the cross-sectional nature of the research design does not allow to properly evaluate the dynamical nature of the stress process and, consequently, to establish causal relations between the analyzed variables. Future longitudinal research or studies using structural equation models could analyze the extent to which students’ psychological well-being promotes more functional coping with academic stress and even contemplate the existence of a bidirectional relationship between these variables. Second, this study did not analyze the role played by some important components of the transactional model of stress such as academic stressors or students’ cognitive appraisal of stressful achievement events (see Skinner and Brewer, 2002). Thus, future works should analyze the interaction between academic demands, psychological well-being, cognitive appraisal, and coping strategies.

Third, although our sample comprised a large number of students (1,072), all of them were from the University of A Coruña, thus limiting the possible generalization of the results to the overall university population. Therefore, future research should corroborate our results with university students from other geographical and cultural contexts.

Fourth, most of the subjects in our sample (almost 70%) were female. Given that the study included majors in all fields of knowledge, we cannot consider this a limitation but rather an indicator of the university landscape today. Overall, we believe that a male sample that was quantitatively more representative could have enabled a deeper analysis regarding the causes and consequences that underlie the gender differences in coping with stress. Thus, we understand that this issue could be a potential line of future research.

A fifth limitation is the use of self-report measures as exclusive data collection method because it can lead to response bias. In future research, combining methodologies to include classroom observations, surveys, and student interviews, would greatly increase our understanding of students’ PL and personal strengths and their ways to manage academic stress. Finally, the limitations of the instrument used to measure coping should be noted. Although the three above-mentioned strategies (positive reappraisal, support-seeking, and planning) constitute good exponents of adaptive coping in classrooms (Skinner et al., 2013), we understand that a more extensive classification of strategies, including both adaptive and dysfunctional strategies, would provide a broader and more comprehensive perspective on their relationship with psychological well-being. In this regard, future work should analyze the possible protective role of psychological well-being compared to markedly undesirable strategies, for example, avoidance or rumination.

## Author Contributions

CF and MF collect data, data analysis, writing the paper. AV writing the paper. JN and GV data analysis, writing the paper.

## Conflict of Interest Statement

The authors declare that the research was conducted in the absence of any commercial or financial relationships that could be construed as a potential conflict of interest.

## References

[B1] Bhullar N. Hine D. W. Phillips W. J. (2014). Profiles of psychological well-being in a sample of Australian university students. *Int. J. Psychol.* 49 288–294. 10.1002/ijop.12022 24990640

[B2] Bryden C. I. Field A. M. Francis A. J. P. (2015). Coping as a mediator between negative life events and eudaimonic well-being in female adolescents. *J. Child Fam. Stud.* 24 3723–3733. 10.1007/s10826-015-0180-0

[B3] Cabanach R. G. Fariña F. Freire C. González P. Ferradás M. M. (2013). Diferencias en el afrontamiento del estrés en estudiantes universitarios hombres y mujeres [Differences in coping between men and women in university studies]. *Eur. J. Educ. Psychol.* 6 19–32.

[B4] Cabanach R. G. Millán P. Freire C. (2009). El afrontamiento del estrés en estudiantes de ciencias de la salud. Diferencias entre hombres y mujeres [Coping strategies in university students of Health Sciences. Differences according to sex]. *Aula Abierta* 37 3–10.

[B5] Cabanach R. G. Souto A. Freire C. Ferradás M. M. (2014). Relaciones entre autoestima y estresores percibidos en estudiantes universitarios [Links between self-esteem and perceived stressors in university students]. *Eur. J. Educ. Psychol.* 7 43–57. 10.1989/ejep.v7i1.151

[B6] Cabanach R. G. Valle A. Rodríguez S. Piñeiro I. Freire C. (2010). Escala de afrontamiento del estrés académico (A-CEA) [The coping scale of academic stress questionnaire (A-CEA)]. *Rev. Iber. Psicol. Salud* 1 51–64.

[B7] Carter M. R. Kelly R. K. Montgomery M. Cheshire M. (2013). An innovate approach to health promotion experiences in community health nursing: a university collaborative partnership. *J. Nurs. Educ.* 52 108–111. 10.3928/01484834-20130121-04 23330668

[B8] Carver C. S. Scheier M. F. (1999). “Stress, coping, and self-regulatory processes,” in *Handbook of Personality: Theory and Research*, 2nd Edn, eds Pervin L. A. John O. P. (New York, NY: Guilford Press), 553–575.

[B9] Cassaretto M. Chau C. Oblitas H. Valdez N. (2003). Estrés y afrontamiento en estudiantes de psicología [Stress and coping in students of psychology]. *Rev. Psicol.* 21 363–392.

[B10] Cohen J. (1988). *Statistical Power Analysis for the Behavioral Sciences.* Hillsdale, NJ: Erlbaum.

[B11] Cohen S. Edwards J. R. (1989). “Personality characteristics as moderators of the relationship between stress and disorder,” in *Advances in the Investigation of Psychological Stress. Wiley Series on Health Psychology/Behavioral Medicine*, ed. Neufeld R. W. J. (Oxford: John Wiley & Sons), 235–283.

[B12] Corp I. B. M. (2012). *SPSS STATISTICS for Windows, Version 21.0.* Armonk, NY: IBM Corp.

[B13] Díaz D. Rodríguez-Carvajal R. Blanco A. Moreno-Jiménez B. Gallardo I. Valle C. (2006). Adaptación española de las escalas de bienestar psicológico de Ryff [Spanish adaptation of the psychological well-being scales (PWBS). *Psicothema* 18 572–577. 17296089

[B14] Doron J. Stephan Y. Boiché J. Le Scanff C. (2009). Coping with examinations: exploring relationships between students’ coping strategies, implicit theories of ability, and perceived control. *Br. J. Educ. Psychol.* 79 515–528. 10.1348/978185409X402580 19187577

[B15] Dyson R. Renk K. (2006). Freshmen adaptation to university life: depressive symptoms, stress, and coping. *J. Clin. Psychol.* 62 1231–1244. 10.1002/jclp.20295 16810671

[B16] Endler N. S. Kantor L. Parker J. D. A. (1994). State-trait coping, state-trait anxiety and academic performance. *Pers. Indiv. Dif.* 16 663–670. 10.1016/0191-8869(94)90208-9

[B17] Endler N. S. Parker J. D. (1990). Multidimensional assessment of coping: a critical evaluation. *J. Pers. Soc. Psychol.* 58 844–854. 10.1037//0022-3514.58.5.844 2348372

[B18] Feldman L. Gonçalves L. Chacón-Puignau G. Zaragoza J. Bagés N. de Pablo J. (2008). Relaciones entre estrés académico, apoyo social, salud mental y rendimiento académico en estudiantes universitarios venezolanos [Relationships between academic stress, social support,mental health and academic performance in Venezuelan university students]. *Univ. Psychol.* 7 739–751.

[B19] Fernández-González L. González-Hernández A. Trianes M. V. (2015). Relationships between academic stress, social support, optimism-pesimism and self-esteem in college students. *Electron. J. Res. Educ. Psychol.* 13 111–130. 10.14204/ejrep.35.14053

[B20] Figueroa M. I. Contini M. Lacunza A. B. Levín M. Estévez A. (2005). Las estrategias de afrontamiento y su relación con el nivel de bienestar psicológico: un estudio con adolescentes de nivel socioeconómico bajo de Tucumán (Argentina) [The coping strategies and its relation with the level of psychological well-being. A research with adolescents of low socioeconomic level of Tucuman (Argentina)]. *An. Psicol.* 21 66–72.

[B21] Fisher S. (1984). *Stress and Perception of Control.* London: Lawrence Erlbaum.

[B22] Folkman S. Lazarus R. S. (1985). If it changes it must be a process: study of emotion and coping during three stages of a college examination. *J. Pers. Soc. Psychol.* 48 150–170. 10.1037/0022-3514.48.1.150 2980281

[B23] Freire C. Ferradás M. M. Núñez J. C. Valle A. (2016). Estructura factorial de las escalas de bienestar psicológico de Ryff en estudiantes universitarios [The factorial structure of Ryff’s psychological well-being scales in university students]. *Eur. J. Educ. Psychol.* 9.

[B24] Freire C. Ferradás M. M. Regueiro B. Piñeiro I. Rodríguez S. Valle A. (2015). Propósitos vitales y afrontamiento del estrés en estudiantes universitarios [Purposes in life and coping in university students]. *Rev. Psicol. Educ. Cult.* 19 42–54.

[B25] Garlow S. J. Rosenberg J. Moore J. D. Haas A. P. Koestner B. Hendin H. (2008). Depression, desperation, and suicidal ideation in college students: results from the American foundation for suicide prevention college screening project at emory university. *Depress. Anxiety* 25 482–488. 10.1002/da.20321 17559087

[B26] González M. T. Landero R. (2007). Factor structure of the perceived stress scale (PSS) in a sample from Mexico. *Span. J. Psychol.* 10 199–206. 10.1017/S1138741600006466 17549893

[B27] González R. Montoya I. Casullo M. M. Bernabéu J. (2002). Relación entre estilos y estrategias de afrontamiento y bienestar psicológico en adolescentes [Relationship between coping strategies and psychological well-being in adolescents]. *Psicothema* 14 363–368.

[B28] González-Torres M. C. Artuch R. (2014). Profiles of resilience and coping strategies at university: contextual and demographic variables. *Electron. J. Res. Educ. Psychol.* 12 621–648. 10.14204/ejrep.34.14032

[B29] Gustems-Carnicer J. Calderón C. (2013). Coping strategies and psychological well-being among teacher education students. *Eur. J. Psychol. Educ.* 28 1127–1140. 10.1007/s10212-012-0158-x

[B30] Hipp J. R. Bauer D. J. (2006). Local solutions in the estimation of growth mixture models. *Psychol. Methods* 11 36–53. 10.1037/1082-989X.11.1.36 16594766

[B31] Kraag G. Zeegers M. P. Kok G. Hosman C. Abu-Saad H. H. (2006). School programs targeting stress management in children and adolescents: a meta-analysis. *J. Sch. Psychol.* 44 449–472. 10.1016/j.jsp.2006.07.001

[B32] Lanza S. T. Flaherty B. P. Collins L. M. (2003). “Latent class and latent transition analysis,” in *Handbook of Psychology: Research Methods in Psychology*, eds Schinka J. A. Velicer W. F. (Hoboken, NJ: Wiley), 663–685.

[B33] Lazarus R. S. (1999). *Stress and Emotion.* New York, NY: Springer.

[B34] Lazarus R. S. Folkman S. (1984). *Stress, Appraisal, and Coping.* New York, NY: Springer.

[B35] Little R. J. Rubin D. B. (1987). *Statistical Analysis with Missing Data.* New York, NY: Wiley.

[B36] Lo Y. Mendell N. R. Rubin D. B. (2001). Testing the number of components in a normal mixture. *Biometrika* 88 767–778.

[B37] Loukzadeh Z. Bafrooi N. M. (2013). Association of coping style and psychological well-being in hospital nurses. *J. Caring Sci.* 2 313–319. 10.5681/jcs.2013.037 25276740 PMC4134144

[B38] Loureiro E. McIntyre T. Mota-Cardoso R. Ferreira M. A. (2008). A relação entre o stress e os estilos de vida nos estudantes de medicina da faculdade de medicina do Porto [The relationship between stress and life-style of students at the faculty of medicine of porto]. *Acta Med. Port.* 21 209–214. 18674412

[B39] Lumley M. A. Provenzano K. M. (2003). Stress management through written emotional disclosure improves academic performance among college students with physical symptoms. *J. Educ. Psychol.* 95 641–649. 10.1037/0022-0663.95.3.641

[B40] Martin A. J. Marsh H. (2009). Academic resilience and academic buoyancy: multidimensional and hierarchical conceptual framing of causes, correlates and cognate constructs. *Oxford Rev. Educ.* 35 353–370. 10.1080/03054980902934639

[B41] Martín M. D. Jiménez M. P. Fernández-Abascal E. G. (1997). Estudio sobre la escala de estilos y estrategias de afrontamiento (E3A) [A study base on the coping styles and strategies scale (E3A)]. *Rev. Electr. Motiv. Emocion* Retrieved from http://reme.uji.es/articulos/agarce4960806100/texto.html

[B42] Matheny K. B. Roque-Tovar B. E. Curlette W. L. (2008). Perceived stress, coping resources, and life satisfaction among U.S. and Mexican college students: a cross-cultural study. *Ann. Psicol.* 24 49–57.

[B43] Maxwell S. E. Delaney H. D. (1993). Bivariate and median splits and spurious statistical significance. *Psychol. Bull.* 113 181–190. 10.1037/0033-2909.113.1.181

[B44] Mayordomo T. Meléndez J. C. Viguer P. Sales A. (2015). Coping strategies as predictors of well-being in youth adult. *Soc. Indic. Res.* 122 479–489. 10.1007/s11205-014-0689-4

[B45] Michie F. Glachan M. Bray D. (2001). An evaluation of factors influencing the academic self-concept, self-esteem and academic stress for direct and re-entry students in Higher Education. *Educ. Psychol.* 21 455–472. 10.1080/01443410120090830

[B46] Miquelon P. Vallerand R. J. (2008). Goal motives, well-being, and physical health: an integrative model. *Can. Psychol.* 49 241–249. 10.1037/a0012759

[B47] Moshki M. Amiri M. Khosravan S. (2012). Mental health promotion of Iranian university students: the effect of self-esteem and health locus of control. *J. Psychiatr. Ment. Health Nurs.* 19 715–721. 10.1111/j.1365-2850.2011.01806.x 22074491

[B48] Muthén L. K. Muthén B. O. (1998–2012). *Mplus User’s Guide*, 6th Edn Los Angeles, CA: Muthén and Muthén.

[B49] Otero-López J. M. Villardefrancos E. Castro C. Santiago M. J. (2014). Stress, positive personal variables and burnout: a path analytic approach. *Eur. J. Educ. Psychol.* 7 95–106. 10.1989/ejep.v7i2.182

[B50] Park C. L. Adler N. E. (2003). Coping style as a predictor of health and well-being across the first year of medical school. *Health Psychol.* 22 627–631. 10.1037/0278-6133.22.6.627 14640860

[B51] Parsons A. Frydenberg E. Poole C. (1996). Overachievement and coping strategies in adolescent males. *Br. J. Educ. Psychol.* 66 109–114. 10.1111/j.2044-8279.1996.tb01180.x 8901172

[B52] Portocarrero M. Bernardes M. L. (2013). Roads to positive self-development: styles of coping that predict well-being. *Int. J. Dev. Educ. Psychol.* 1 383–392.

[B53] Putwain D. W. Connors L. Symes W. Douglas-Osborn E. (2012). Is academic buoyancy anything more than adaptive coping? *Anxiety Stress Coping* 25 349–358. 10.1080/10615806.2011.582459 21644112

[B54] Rodríguez J. Agulló E. (1999). Estilos de vida, cultura, ocio y tiempo libre de los estudiantes universitarios [Lifestyles, culture, leisure and free time of university students]. *Psicothema* 11 247–259.

[B55] Romero A. Cruz S. Gallardo C. Peñacoba C. (2013). Cómo promocionar la salud la salud y el bienestar en la comunidad universitaria. Universidad Rey Juan Carlos, universidad saludable [How to promote health and well-being for the entire university community. Rey Juan Carlos University, healthy university]. *Rev. Iber. Psicol. Salud* 4 49–64.

[B56] Ryan R. M. Deci E. L. (2000). Self-determination theory and the facilitation of intrinsic motivation, social development and well-being. *Am. Psychol.* 55 68–78. 10.1037/0003-066X.55.1.68 11392867

[B57] Ryff C. D. (1989). Happiness is everything, or is it? Explorations on the meaning of psychological well-being. *J. Pers. Soc. Psychol.* 57 1069–1081. 10.1037/0022-3514.57.6.1069

[B58] Salanova M. Martínez I. M. Bresó E. Llorens S. Grau R. (2005). Bienestar psicológico en estudiantes universitarios: facilitadores y obstaculizadores del desempeño académico [Psychological well-being among university students: facilitators and obstacles of academic performance]. *Ann. Psicol.* 21 170–180.

[B59] Sheldon K. M. Lyubomirsky S. (2006). How to increase and sustain positive emotion: the effects of expressing gratitude and visualizing best possible selves. *J. Posit. Psychol.* 1 73–82. 10.1080/17439760500510676

[B60] Skinner E. A. Edge K. Altman J. Sherwood H. (2003). Searching for the structure of coping: a review and critique of category systems for classifying ways of coping. *Psychol. Bull.* 129 216–269. 10.1037/0033-2909.129.2.216 12696840

[B61] Skinner E. A. Pitzer J. Steele J. (2013). Coping as part of motivational resilience in school. A multidimensional measure of families, allocations, and profiles of academic coping. *Educ. Psychol. Meas.* 73 803–835. 10.1177/0013164413485241

[B62] Skinner N. Brewer N. (2002). The dynamics of threat and challenge appraisals prior to stressful achievement events. *J. Pers. Soc. Psychol.* 83 678–692. 10.1037/0022-3514.83.3.678 12219862

[B63] Springer K. W. Hauser R. M. (2006). An assessment of the construct validity of Ryff’s Scales of Psychological Well-being: method, mode and measurement effects. *Soc. Sci. Res.* 35 1080–1102. 10.1016/j.ssresearch.2005.07.004

[B64] Syed M. Seiffge-Krenke I. (2015). Change in ego development, coping, and symptomatology from adolescence to emerging adulthood. *J. Appl. Dev. Psychol.* 41 110–119. 10.1016/j.appdev.2015.09.003

[B65] Taylor S. E. (1991). *Seamos Optimistas. Ilusiones Positivas [Let’s be Optimistic. Positive Illusions].* Barcelona: Martínez-Roca.

[B66] Tomás J. M. Sancho P. Meléndez J. C. Mayordomo T. (2012). Resilience and coping as predictors of general well-being in the elderly: a structural equation modelling approach. *Aging Ment. Health* 16 317–326. 10.1080/13607863.2011.615737 22292552

[B67] Viñas F. González M. García Y. Malo S. Casas F. (2015). Los estilos y estrategias de afrontamiento y su relación con el bienestar personal en una muestra de adolescentes [Coping strategies and styles and their relationship to personal well-being in a sample of adolescents]. *Ann. Psicol.* 31 226–233. 10.6018/analesps.31.1.163681

[B68] Zajacova A. Lynch S. M. Espenshade T. J. (2005). Self-efficacy, stress, and academic success in college. *Res. High. Educ.* 46 677–706. 10.1007/s11162-004-4139-z

[B69] Zeidner M. (1995). Adaptive coping with test situations: a review of the literature. *Educ. Psychol.* 30 123–133. 10.1207/s15326985ep3003_3

